# LncRNA BIRF Promotes Brain Ischemic Tolerance Induced By Cerebral Ischemic Preconditioning Through Upregulating GLT-1 via Sponging miR-330-5p

**DOI:** 10.1007/s12035-022-02841-3

**Published:** 2022-04-22

**Authors:** Shichao Li, Lingyan Zhang, Jiajie Lin, Achou Su, Xiyun Liu, Jingge Zhang, Xiaohui Xian, Yuyan Hu, Wenbin Li, Shaoguang Sun, Min Zhang

**Affiliations:** 1 Key Laboratory of Critical Disease Mechanism and intervention of Hebei Province, Department of Pathophysiology, Hebei Medical University, Shijiazhuang, China; 2 Key Laboratory of Medical Biotechnology of Hebei Province, Hebei Medical University, Department of Biochemistry and Molecular Biology, Cardiovascular Medical Science Center, Shijiazhuang, China

**Keywords:** Long non-coding RNAs, MicroRNAs, Competing endogenous RNAs, GLT-1, Ischemic preconditioning, Stroke

## Abstract

Long noncoding RNAs (lncRNAs) play an important regulatory role in various diseases. However, the role of lncRNAs in brain ischemic tolerance (BIT) induced by cerebral ischemic preconditioning (CIPC) is still unknown. The lncRNA profile of rat cortical astrocytes pretreated with ischemic preconditioning was analyzed by high-throughput sequencing. The results of Cell-Counting Kit-8 (CCK-8) assay showed that a novel lncRNA, NONRATT009133.2, which we referred to as brain ischemia–related factor (BIRF), was highly correlated with BIT. Through bioinformatics analysis, we predicted that BIRF, miR-330-5p, and GLT-1 (also named *Slc1a2*) might constitute a ceRNA regulatory network in the induction of BIT. We found that BIRF was upregulated by CIPC, which promoted GLT-1 expression and BIT induction. BIRF could directly bind to miR-330-5p. Furthermore, miR-330-5p directly targeted GLT-1, and miR-330-5p inhibited both GLT-1 expression and BIT induction in vitro and in vivo. Moreover, BIRF acts as a molecular sponge to competitively bind to miR-330-5p with GLT-1 mRNA, while the miR-330-5p inhibitor reversed all the effects of BIRF siRNA on GLT-1 expression and neuronal vitality. Taken together, our results demonstrate the important roles of the BIRF/miR-330-5p/GLT-1 axis in the induction of BIT by CIPC. BIRF may be a potentially effective therapeutic strategy against stroke injury.

**Supplementary Information:**

The online version contains supplementary material available at 10.1007/s12035-022-02841-3.

## Introduction

Ischemic stroke is a disease with high levels of mortality and disability. Since dead neurons barely regenerate, many researchers focus on how to improve the neuronal tolerance to ischemic injury. Many studies have proved that transient sublethal cerebral ischemia can protect neurons survival from subsequent lethal ischemic insult, which is known as brain ischemic tolerance (BIT), and the transient sublethal ischemia is called cerebral ischemic preconditioning (CIPC) [1]. However, the mechanism of the induction of BIT by CIPC is not very clear.

With the rapid development of high-throughput sequencing and the wide application of bioinformatics, dysregulated transcripts, including microRNAs (miRNAs), long noncoding RNAs (lncRNAs), and circular RNAs (circRNAs), can be explored in many diseases [2, 3]. LncRNAs are a set of RNAs longer than 200 nucleotides with no protein-coding ability. LncRNAs have been shown to be involved in numerous human diseases [4, 5]. The research on lncRNAs involved in regulating cerebral ischemia is drawing increasing attention. Silencing of lncRNA Nespas has been shown to aggravate microglial cell death and neuroinflammation in ischemic stroke [6]. Moreover, lncRNA H19 promotes neuroinflammation in ischemic stroke by driving histone deacetylase 1-dependent M1 microglial polarization [7]. However, whether lncRNAs participate in CIPC-induced BIT remains unknown.

MiRNAs are endogenously expressed small noncoding RNAs with a length of 18–24 nucleotides, which regulate gene expression mainly by binding to the target mRNAs [8, 9]. MiRNAs also participate in the occurrence and development of a variety of diseases. M2 microglia-derived exosomes protect the mouse brain from ischemia-reperfusion injury via exosomal miR-124 [10]. Remote postconditioning ameliorates stroke injury by preventing the upregulation of let-7a and miR-143 [11]. However, the role of miRNA in the induction of BIT by CIPC requires further exploration.

Growing evidence has implicated that many lncRNAs function as competing endogenous RNAs (ceRNAs) to affect ischemic stroke [12]. For example, in the oxygen glucose deprivation (OGD) model of cerebral microvascular endothelial cells, lncRNA MALAT1 has been shown to upregulate SIRT1 expression by binding with miR-200C-3p, this induced autophagy, which promoted the survival of endothelial cells [13]. LncRNA MALAT1 has also been shown to be upregulated in cerebral ischemic injury, where it induced miR-30a inhibition, which increased the expression of Beclin 1, and subsequently promoted autophagy and neuronal death [14]. Nevertheless, it is needed to be studied whether the ceRNA mechanism participates in the induction of BIT by CIPC.

It is well known that glutamate is an important excitatory neurotransmitter in the central nervous system. Glutamate homeostasis in the central nervous system is mainly maintained by excitatory amino acid transports (EAATs). Five types of high-affinity EAATs have been identified, of which EAAT2, also named glial glutamate transporter-1 (GLT-1), solute carrier family 1 member 2 (*Slc1a2*), is mainly distributed in astrocytes, and is responsible for 90% of glutamate uptake [15–17]. During acute episodes of stroke, rapid elevation of glutamate leads to neuronal injury, which is known as excitotoxicity [18]. Several previous studies, including our own, have demonstrated that GLT-1 upregulation plays a critical role in the induction of BIT by CIPC through the prevention of excessive glutamate gathering and the termination of multiple downstream death signaling cascades [19–23].

In the present study, we focused on whether lncRNAs play an important role in the induction of BIT by regulating GLT-1. To answer this question, we investigated the expression profiling of lncRNAs during the induction of BIT. Based on the fold change and expression abundance of lncRNAs, we selected lncRNA NONRATT009133.2, which we named brain ischemia–related factor (BIRF), for further research. Next, through bioinformatics analysis, we predicted that BIRF, miR-330-5p, and GLT-1 might constitute a ceRNA regulatory network in the induction of BIT. The present study focuses on the mechanism of BIRF/miR-330-5p/GLT-1 axis in the induction of BIT, which may provide a potential prognostic biomarker and therapeutic target for ischemic stroke.

## Materials and Methods

### Animals

Male Wistar rats (8 weeks old, 280–320 g in weight) provided by the Experimental Animal Center of Hebei Medical University were used for experiments in vivo. Rats were housed two per cage and maintained in an air-conditioned room (approximately 20–24 °C) in a 12-h light/12-h dark cycle, with access to food and water ad libitum. Neonatal Wistar rats (within 24 h after birth, gender was not differentiated) were used for experiments in vitro. All the animal studies were approved by the Laboratory Animal Ethical and Welfare Committee of Hebei Medical University (Approval No. IACUC-Hebmu-2020013). In accordance with the guidelines for the care and use of laboratory animals, we tried our best to minimize the number of rats used in the experiments and to relieve rat suffering. To minimize animal suffering, all animals were anesthetized with 2–2.5% isoflurane, and 1% lidocaine was injected around the incision site during surgery. Adult rats were sacrificed by decapitation under inhalation anesthesia with 4–5% isoflurane for the sampling of fresh brains. All efforts were made to minimize the number of animals used in the experiments, we used the same rat brain tissue for thionin staining and TUNEL staining, and we cultured the neurons and astrocytes from cerebral cortex instead of hippocampal CA1 subfield. Male Wistar rats were randomly divided into every group. All drugs used in the present study were purchased from 2019 to 2021.

### Global Brain Ischemia

The global brain ischemic model was established by four-vessel occlusion [24]. Briefly, the bilateral vertebral arteries of the rats were permanently electrocauterized under isoflurane anesthesia (2–2.5%). Two days after the operation, the bilateral common carotid arteries of the rats were exposed under isoflurane and then clamped for a short period of 3 min for CIPC or 8 min for ischemic insult. When CIPC was followed by ischemic insult, the interval between them was 2 days. This protocol has been proven to be effective in inducing brain ischemic tolerance in our previous studies [21, 22]. Only rats whose pupils were obviously dilated, lost consciousness, and the righting reflex disappeared after four-vessel occlusion were used for the following experiments. The body temperature of the rats was maintained at about 37 °C during the operations until the rats recovered. The sham operation included all surgical procedures except for the occlusion of the bilateral common carotid arteries.

### Intracerebroventricular Injection

Lateral ventricular injection was performed 2 weeks before cerebral ischemia. Adeno-associated virus (AAV) encoding short-hairpin RNA targeting BIRF, AAV encoding miR-330-5p, and negative control (NC) (GenePharma, Shanghai, China) were used to infect rat brain tissue. pAAV-U6-EGFP-sh-BIRF, pAAV-U6-EGFP-sh-NC, pAAV-U6-EGFP-miR-330-5p, or pAAV-U6-EGFP-miR-NC (3 ul of 10^12^ VG/mL; GenePharma, Shanghai, China) was stereotaxically injected into the lateral ventricle via a needle with an outer diameter of 0.4 mm connected to a microsyringe. The injection points were located anteroposterior (AP) − 0.8 mm, mediolateral (ML) − 1.50 mm, and dorsoventral (DV) − 3.8 mm to the bregma. The protocol was successfully carried out in our laboratory [22]. The pAAV-sh-BIRF, pAAV-miR-330-5p sequences are available in Table S1.

### Neuropathological Evaluation

Neuropathological evaluation was performed 7 days after cerebral ischemia in the same manner as we followed before [20]. Briefly, the rats were perfused with 4% paraformaldehyde under anesthesia with isoflurane. The brain was quickly collected, and a 1 to 4 mm postoptic chiasma was taken. The paraffin-embedded brain tissues were coronally sliced at a thickness of 5 μm and stained with thionin to observe the degree of delayed neuronal injury. The number of surviving pyramidal neurons in the CA1 hippocampus within a linear length of 1 mm was considered as neuronal density, which was counted in the manner previously described [25].

### Cell Culture and Treatments

Primary astrocytes and neurons were cultured according to the previously described methods [20]. It is well known that cerebral cortical neurons are just as sensitive to ischemia as hippocampal CA1 neurons, and cerebral cortex is large to get more neurons and astrocytes than CA1 hippocampus. Therefore, we cultured the neurons and astrocytes from cerebral cortex to minimize the number of animals used for the experiments. Briefly, cerebral cortices were taken from neonatal Wistar rats (< 24 h) and digested with 2 mg/mL papain (Acros, USA) and 42 μg/mL deoxyribonuclease (Sigma, USA) at 37 °C for 30 min. The cells were then mechanically separated and suspended in Dulbecco’s modified Eagle’s medium (DMEM)/F12 (Gibco, USA) containing 10% fetal bovine serum (Gemini, USA) and 1% penicillin-streptomycin (Gibco, USA). As for the astrocytes, the cell suspension was seeded into cell culture flasks, and then they were incubated in a humidified atmosphere of 5% CO_2_ and 95% air at 37°C. Following 24-h incubation, the medium was changed to fresh DMEM/F12 containing 10% fetal bovine serum and 1% penicillin-streptomycin, and then renewed every 3 days. On the 6th–8th day, astrocytes were sub-cultured and plated at a density of 5 × 10^5^ cells/cm^2^ on 14 mm glass coverslips in 24-well plates for immunocytochemical staining, or on 25 mm glass coverslips in 6-well plates for western blotting or qRT-PCR.

For primary neurons, the cell suspension was seeded on poly-D-lysine-coated plates with paraffin dots adhered to the bottom and incubated in a humidified atmosphere of 5%CO_2_ and 95% air at 37 °C, the cell density was 1 × 10^6^ cells/cm^2^ in 6-well plates and 6 × 10^5^ cells/cm^2^ in 24-well plates. After 4 h, the medium was replaced with neurobasal medium (Gibco, USA) supplemented with 2% B-27 (Gibco, USA) and 1% penicillin-streptomycin (Gibco, USA). Half the volume of medium was replaced every 2 days.

After the astrocytes were sub-cultured and plated for 2–3 days, coverslips containing astrocytes were placed on top of the neuron cultures which were cultured for approximately 6–8 days. The astrocytes on coverslips were separated from neurons by paraffin dots, and both cell types were immersed in the neurobasal medium supplemented with 2% B-27 and 1% penicillin-streptomycin. Co-cultures were incubated in a normoxic incubator for 48 h and then used for subsequent OGD or IPC studies.

Astrocyte marker GFAP, and neuron marker NSE were done to confirm the specificities of cell culture for astrocytes and neurons (Fig. S1).

### OGD and IPC Model

OGD was used to simulate cerebral ischemia after co-culture of astrocytes and neurons. OGD was performed as previously described [20]. Co-cultures were rinsed twice with 1*PBS (Gibco, USA) and then incubated in glucose-free DMEM (Gibco, USA) in a hypoxic incubator (model 3131, Thermo Fisher Scientific, USA) containing 94% N_2_, 1% O_2,_ and 5% CO_2_ for 45 min or 4 h in the IPC group or the OGD group, respectively. Then, cells were placed into normal medium under normoxic conditions for another 24 h, which was considered reoxygenation. In the control group, the co-cultures were always cultured with maintenance medium under normoxic conditions. For the IPC + OGD group, IPC was followed by lethal OGD, with an interval of 24 h.

### High-Throughput Sequencing and Bioinformatics Analysis

Astrocytes were sent to the Sinotech Genomics Corporation for high-throughput sequencing based on the Illumina Novaseq 6000 sequencing platform. Through the sequencing results library of lncRNAs and sequence alignment produced by Sinotech Genomics (Shanghai, China), we obtained a diagram describing the lncRNA-miRNA relationship. Subsequently, we searched for mRNAs that had potential binding sites with miRNAs using the miRWalk database. For these mRNAs, we performed Gene Ontology (GO) categories analysis derived from Gene Ontology (www.geneontology.org) and pathway analysis based on the latest Kyoto Encyclopedia of Genes and Genomes (http://www.genome.jp/kegg) database. *P* < 0.05 was the threshold for statistical significance. The result of lncRNA high-throughput sequencing (RNA-seq) have been uploaded to GEO database (GSE185931, https://www.ncbi.nlm.nih.gov/geo/query/acc.cgi?acc=GSE185931), and released on November 1, 2021.

### siRNA and Cell Transfection

BIRF siRNA and its negative control (siRNA NC), miR-330-5p mimics and its negative control (mimics NC), and miR-330-5p inhibitor and its negative control (inhibitor NC) (GenePharma, Shanghai, China) were synthesized and transfected with a Lipofectamine 3000 Transfection Kit (Invitrogen, CA, USA) according to the manufacturer’s instructions when astrocytes were grown to a 50–60% confluence on the slide. After 24-h transfection, astrocytes were co-cultured with neurons, and after 24-h co-culture, the next experiment was performed. The siRNAs, mimics, and inhibitors sequences are available in Tables S2–S4.

### Hoechst 33342 and Propidium Iodide (Hoechst-PI) Staining

Neuronal death rate was investigated by Hoechst 33342 (Solarbio, Beijing, China) and propidium iodide (PI, Sigma, USA) staining. After OGD, the co-cultures regained oxygen and glucose supply for 24 h. Then, the coverslips containing astrocytes were discarded, and the neurons were washed twice on 24-well plates with 1× PBS and subsequently stained with Hoechst 33342 and PI at a concentration of 10 μg/mL at 37 °C for 15 min. Hoechst 33342 could stain all nuclei of neurons that appeared dark blue, and PI could enter the neurons when membrane integrity was destroyed and exhibited red fluorescence. The neurons were observed and photographed under an inverted fluorescence microscope (Nikon, ECLIPSE Ti, Japan). The neuronal death rate was determined by the formula: [(necrotic neurons + apoptotic neurons)/total neurons] × 100%.

### CCK-8 Assay

Neuronal viability was detected by the Cell Counting Kit-8 (CCK-8, MCE, USA) assay following the manufacturer’s instructions. Twenty-four hours after OGD/reoxygenation, the coverslips containing astrocytes were discarded, and the medium for the neurons was replaced with fresh medium including CCK-8 and then incubated for a further 2.5 h at 37 °C. The absorbance of each well was measured at 450 nm using a microplate reader (BioTek, SYNERGY/HT, USA). The percentage of viable neurons was calculated using the following formula: neuronal viability (%) = A450 (sample)/A450 (control) × 100.

### Dual-Luciferase Reporter Assays

Dual-luciferase reporter gene analysis was used to determine the interaction targets between lncRNA and miRNA, and miRNA and mRNA [26]. The supposed binding sites of miR-330-5p with BIRF and the GLT-1-CDS fragment were cloned by PCR and inserted into a psiCHECK™-2 Vector (Promega, Madison, WI, USA) to create a luciferase reporter vector (lncRNA-BIRF-wt and GLT-1-wt) (Sangon Biotech, Shanghai, China). Corresponding mutants (lncRNA-BIRF-mut and GLT-1-mut) (Sangon Biotech, Shanghai, China) were constructed by genetic alteration of the supposed binding sites. Subsequently, HEK-293T cells (Cat No.: CL-0005), which were acquired from Procell Life Science and Technology Co., Ltd., were transfected with psiCHECK™-2 vectors and miRNA mimics by Lipofectamine 3000. Relative luciferase activities were evaluated using a Dual-Luciferase® Reporter Assay System (Promega, USA) 24 h after transfection. The renilla luciferase activity was normalized by the firefly luciferase activity.

### RNA FISH

The subcellular localization of lncRNA-BIRF was detected by fluorescence in situ hybridization (FISH) [27]. The lncRNA-BIRF probe and its negative control were synthesized (RIBOBIO, Guangzhou, China), and a fluorescence in situ hybridization kit (RIBOBIO, Guangzhou, China) was used to perform this experiment. Briefly, coverslips containing astrocytes were fixed with paraformaldehyde, and then Triton X-100 was used to permeabilize the cells. Triton X-100 was removed by washing, and the coverslips were sealed with pre-hybridization solution. Subsequently, the coverslips were mixed with probe hybridization solution and incubated overnight at 37 °C. Finally, DAPI staining was performed, and the slices were sealed with anti-fluorescence quenching-sealing agent (Solarbio, Beijing, China) and observed under a laser scanning confocal microscope (OLYMPUS, Japan).

### Immunocytochemistry Assay

Astrocytes cultured on 24-well coverslips were removed from the culture medium. The coverslips were cleaned with 1× PBS twice, and endogenous peroxidase blocker in the Universal SP Kit (Streptomyces Ovoaltin-biotin assay system) (ZSGB-BIO, Origene, Beijing, China) was added to the coverslips for 15 min, after cleaning them with 1× PBS and sealing them with anti-goat serum for 30 min; primary antibody against GLT-1 (1:200, Cat No.: #AB1783, Lot No.: #3305889, Millipore, USA) was added to coverslips at 4 °C overnight. On the second day, the coverslips were sealed with biotinylated labeled goat anti-guinea pig IgG(H+L) (1:3000, Cat No.: 5260-0440, Lot No.: XH051, KPL, USA) for GLT-1 (the second antibody) at 37 °C for 1 h and washed. Then, horseradish peroxidase-conjugated streptavidin in the Universal SP Kit was added at 37 °C for 1 h and washed. Then, DAB kit was used for color rendering. Finally, the slices were sealed with neutral gum and observed under a microscope (OLYMPUS, DP80, Japan).

### TUNEL Staining

Terminal-deoxynucleotidyl transferase-mediated nick end labeling (TUNEL) assay (KeyGen Biotech, Nanjing, China) was conducted to evaluate apoptosis. Paraffin sections were prepared by conventional methods. After the sections were dewaxed with xylene, hydrated with ethanol gradient, and permeated with proteinase K, the samples were treated with TdT enzyme reaction solution. Finally, streptavidin-fluorescein labeling solution was dropped onto the slide, and the slide was placed into a wet box for incubation at 37 °C for 30 min. We used anti-fluorescence quenching-sealing agent (containing DAPI) to seal the slide (Solarbio, Beijing, China). Finally, we observed and photographed the samples under a microscope (OLYMPUS, DP80, Japan).

### qRT-PCR

Total RNA was extracted from brain tissues and cells using TRIzol reagent (Life Technologies Corporation, Carlsbad, CA, USA). RNA concentrations were detected, and the quality was determined at 260/280 nm absorbance using a microplate reader (BioTek, USA). ChamQ Universal SYBR qRCR Master Mix (Vazyme Biotech Co., Ltd., Nanjing, China) was applied to determine lncRNAs, GLT-1 mRNA, and GAPDH expression. All-in-One™ miRNA qRT-PCR Detection System 2.0 U (GeneCopoeia, Inc., Guangzhou, China) was used to determine miR-330-5p and U6 expression. The fold change was equivalent to the relative expression normalized to endogenous control (2^−ΔΔCt^). GAPDH and U6 were used as internal controls. Each qRT-PCR was performed in at least triplicate to verify the stability and repeatability of the results. The primer sequences are available in Tables S5 and S6.

### Western Blot

Protein levels of GLT-1, Caspase-3, Bcl-2, Bax, and β-actin were examined by western blot. Briefly, astrocytes, neurons, or CA1 hippocampus were harvested and lysed using the RIPA buffer, which contained protease inhibitors (Roche, Germany) and phosphatase inhibitors (Roche, Germany) to obtain total protein. Lysates were centrifuged at 12000 rpm/min for 15 min at 4 °C, and the supernatant was collected. The concentrations of protein in the supernatant were measured using the BCA Protein Assay kit (Solarbio, Beijing, China). Proteins were separated on a 12% SDS-polyacrylamide gel and electrophoretically transferred to polyvinylidene difluoride (PVDF) membranes (Millipore, Germany). The membranes were blocked in 5% bovine serum albumin (Biofroxx, Germany) in TBST at 37 °C for 1 h and then respectively incubated with primary antibodies against GLT-1 (1:3000, Cat No.: #AB1783, Lot No.: #3305889, Millipore, USA), Caspase-3 (1:1000, Cat No.: GTX110543, Lot No.: 42879, GeneTex, Taiwan, China), Bcl-2 (1:500, Cat No.: GTX100064, Lot No.: 42970, GeneTex, Taiwan, China), Bax (1:2000, Cat No.: ET1603-34, Lot No.: HN0921, HUABIO, Hangzhou, China), and β-actin (1:3000, Cat No.: 20536-1-AP, Proteintech, USA) at 4 °C for 16 h. Then, after washing with TBST, they were subsequently incubated with the secondary antibodies to either peroxidase-conjugated Affinipure Goat Anti-Rabbit IgG(H+L) (1:3000, Cat No.: SA00001-2, Lot No.: 20000258, Proteintech, USA) for caspase-3, Bcl-2, Bax, β-actin, or biotinylated labeled goat anti-guinea pig IgG (H+L) (1:3000, Cat No.: 5260-0440, Lot No.: XH051, KPL, USA) for GLT-1 at 37 °C for 1 h. After that, the membranes were washed with TBST. Only the membrane for GLT-1 was subsequently incubated with HRP-conjugated streptavidin (1:3000, Cat No.: 43-4323, Lot No.: 1513798 A, KPL, USA) at 37 °C for 1 h. Followed by washing with TBST, protein bands were detected by an enhanced chemiluminescence kit (ECL kit; Monad Biotech Co., Ltd., Suzhou, China) under a chemiluminescence imaging analysis system (Amersham Imager 600, GE, CT, USA). The integral optical density (IOD) of each band was performed using analysis software (Alpha Image 2200, Alpha, USA), and β-actin was used to confirm equal sample loading and normalization of the data.

### Statistical Analyses

SPSS Statistics 23 (IBM, Chicago, IL, USA) was used for statistical analysis, and data were expressed as mean ± SD (standard deviation), which passed normality tests and variance similar tests. For in vitro experiments, we pooled the samples from six culture wells and repeated the experiments at least three times. We evaluated the data using Student’s *t*-test, and multiple comparisons were performed through one-way ANOVA, followed by the Student–Newman–Keuls test involving all groups. *P* < 0.05 was considered to be significant. GraphPad Prism 8 and Adobe Illustrator 2020 were used to create the artwork.

## Results

### BIRF Was Significantly Elevated in Astrocytes After Ischemic Preconditioning, and the Possible ceRNA Relationship Between BIRF, miR-330-5p, and GLT-1

**Fig. 1:**
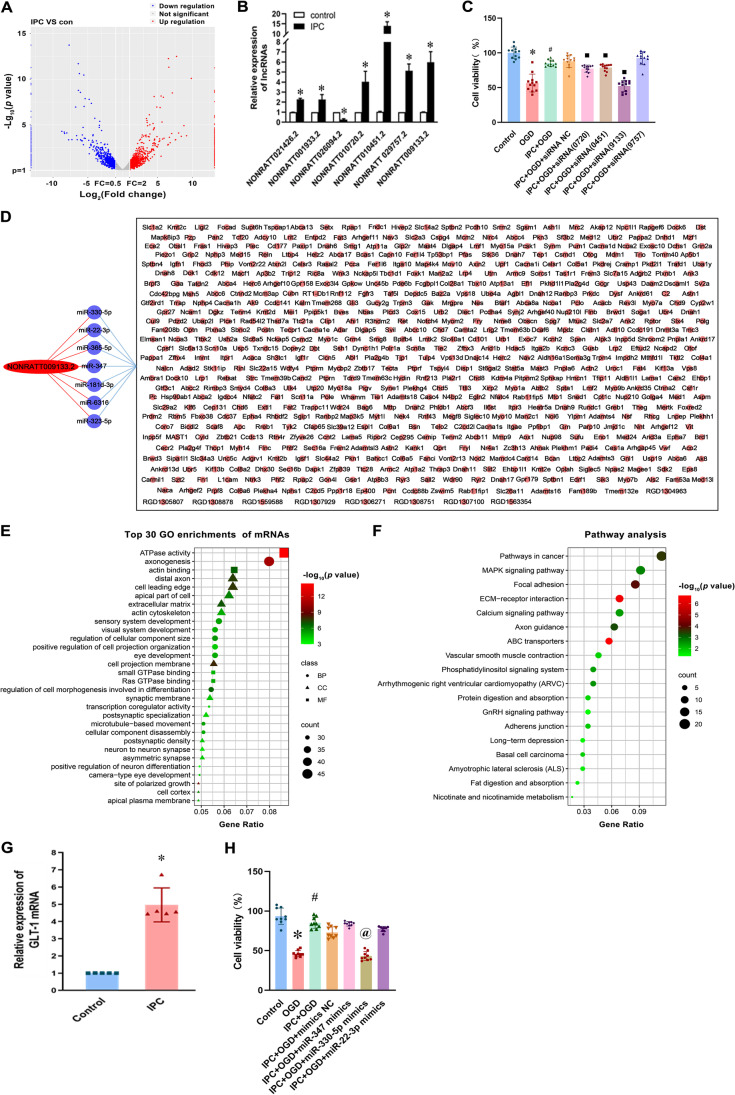
Verification of high-throughput sequencing for lncRNA in rat cortical astrocytes and bioinformatics analysis. **A** Volcanic map of differentially expressed lncRNAs (*n* = 2). **B** qRT-PCR validation for lncRNAs with significant difference (*n* = 7). **C** The effect of knockdown of the lncRNAs on cell viability (*n* = 12). **D** Construction of ceRNA network centered on NONRATT009133.2 (BIRF). Red elliptic node: the upregulated NONRATT009133.2. Blue circular nodes: miRNAs. Pink circular nodes: target genes. **E** Top 30 GO enrichment of mRNAs from the ceRNA network. **F** Pathway analysis of mRNAs from the ceRNA network. **G** qRT-PCR analysis of the expression of GLT-1 (*Slc1a2*) after IPC in co-cultured astrocytes (*n* = 5). **H** The effect of miRNA mimics on cell viability (*n* = 9). Data are presented as the mean ± SD. *P < 0.05 vs. control group; ^#^*P* < 0.05 vs. OGD group; ^■^*P* < 0.05 vs. IPC+OGD+siRNA NC group; ^@^*P* < 0.05 vs. IPC+OGD+mimics NC group

We have previously reported that ischemic preconditioning (IPC)-induced BIT [28]. To further explore the role of astrocytes in the process, in this study, cerebral cortical astrocytes and neurons of rats were co-cultured, the astrocytes of the co-cultured cell model pretreated by IPC (45 min of OGD) were subjected to lncRNA high-throughput sequencing (RNA-seq). The results showed that the lncRNA profile of the IPC group was significantly different from that of the control group (Fig. 1A). Based on the fold change and expression abundance of lncRNAs, seven lncRNAs were selected for qRT-PCR validation. As shown in Fig. 1B, among the seven lncRNAs, NONRATT010720.2, NONRATT010451.2, NONRATT029757.2, and NONRATT009133.2 showed the most significant differences. Therefore, we transfected astrocytes with the siRNAs of these four lncRNAs and performed a CCK8 assay to test their effects on the OGD tolerance of neurons. The result suggested that NONRATT009133.2 siRNA had the greatest impact on OGD tolerance induced by IPC, and was therefore taken forward for further analysis (Fig. 1C). Through the sequencing results library construction and sequence alignment produced by Sinotech Genomics (Shanghai, China), we predicted 7 miRNAs that had the potential to bind to NONRATT009133.2 (Fig. 1D). Then, we used miRWalk (http://mirwalk.umm.uni-heidelberg.de/) to predict the mRNAs that were likely to bind to these 7 miRNAs (Fig. 1D). Red elliptic node represents the upregulated NONRATT009133.2. Blue circular nodes indicate the 7 miRNAs predicted by Sinotech Genomics to be sponged by NONRATT009133.2. Pink circular nodes represent 634 target genes predicted by the miRWalk database to be shared by the 7 miRNAs above. Subsequently, we performed gene ontology (GO) categories analysis and pathway analysis on these mRNAs (Fig. 1E, F). GO and pathway analysis results implied that NONRATT009133.2 is associated with cerebral ischemia-reperfusion injury (Table S7). What is more, *Slc1a2*, which was proven to play a key role in CIPC-induced BIT in our previous studies, is also among these mRNAs. Our previous studies have confirmed that CIPC can upregulate GLT-1 (also named *Slc1a2*) expression and induce BIT [20]. qRT-PCR result shows that GLT-1 was up-regulated after IPC in co-cultured astrocytes (Fig. 1G). Through a literature review, we learned that among the 7 miRNAs, miR-330-5p, miR-347, and miR-22-3p were related to ischemia reperfusion injury [29–31]. We next observed the effects of the 3 miRNAs on OGD tolerance of neurons through transfection of these miRNA mimics into astrocytes. As revealed by the CCK8 assay, we found that miR-330-5p had greater influence on reducing neuronal viability; therefore, we selected miR-330-5p for further research (Fig. 1H).

Thus, we identified three key molecules that are likely to be involved in IPC-induced OGD tolerance, and suggest that NONRATT009133.2/miR-330-5p/GLT-1 (*Slc1a2*) may play a role through a ceRNA mechanism. For convenience, according to the function of NONRATT009133.2, we named it brain ischemia–related factor (BIRF).

### BIRF Silencing Downregulated GLT-1 Expression and Impeded the Induction of OGD Tolerance

**Fig. 2:**
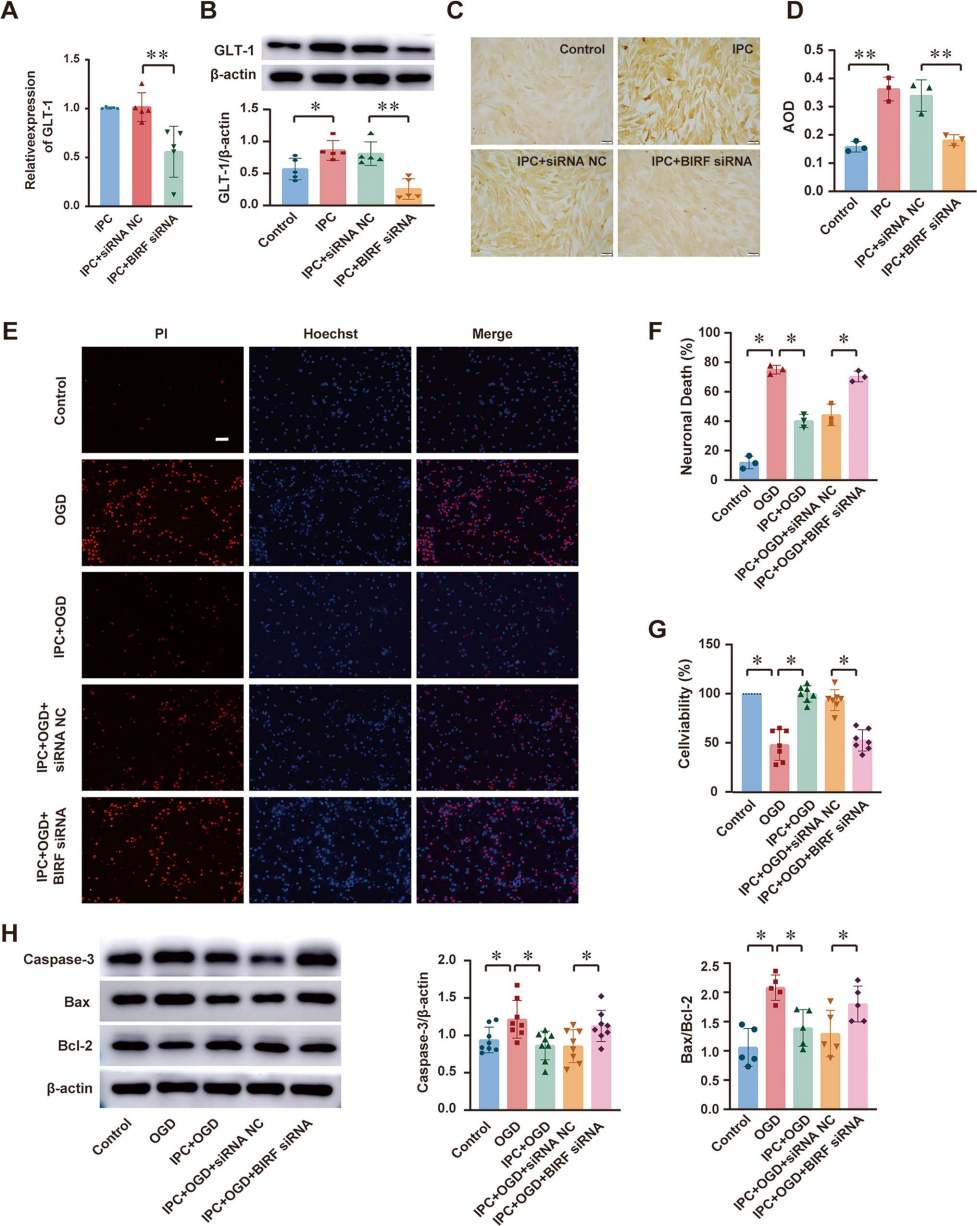
BIRF regulates GLT-1 expression and OGD tolerance in vitro (**A** and **B**) The effect of BIRF siRNA on the expression of GLT-1 mRNA by qRT-PCR (**A**) and protein by western blot (**B**) in astrocytes (*n* = 5). **C**,** D** Representative immunocytochemical staining photomicrographs show the effect of BIRF siRNA on GLT-1 expression in astrocyte (**C**) (scale bar = 50 μm) and its AOD measured using ImageJ software (**D**) (*n* = 3). **E**,** F** Hoechst-PI shows cell death of neurons (**E**) (scale bar = 50 μm) and statistical comparison of neuronal death rate (F) (*n* = 3). **G** CCK-8 assay was conducted to explore the influence of BIRF siRNA on cell viability of neurons (*n* = 7). **H** The effect of BIRF siRNA on caspase-3 (*n* = 8) and Bax/Bcl-2 (*n* = 5) expression in neurons by western blot. Data are presented as the mean ± SD. *P < 0.05, **P < 0.01

To investigate the regulatory mechanism of BIRF in OGD tolerance, the co-cultured astrocytes were transfected with BIRF siRNA 2 days before IPC. The transfection and knockdown efficiency were verified (Fig. S2). We found that BIRF siRNA could inhibit the expression of GLT-1 mRNA (Fig. 2A) and protein (Fig. 2B–D) in astrocytes. Since IPC could induce OGD tolerance though upregulating GLT-1 expression [21, 22], we focused on the impacts of BIRF siRNA on OGD tolerance. We detected the neuronal death rate using Hoechst PI (Fig. 2E, F), and cell viability using the CCK8 assay (Fig. 2G). Lethal OGD (4 h of OGD) led to neuronal death, and IPC treatment before OGD could decrease the neuronal death, but this protective effect of IPC was blocked after transfection of BIRF siRNA (Fig. 2E, F). In addition, the results of the CCK8 assay showed that the impairment of cell viability after OGD was alleviated by IPC, and this change could be abolished by BIRF siRNA (Fig. 2G). Furthermore, western blot was performed to explore the expression of apoptosis-related proteins in neurons (Fig. 2H), and the results showed that IPC+OGD significantly downregulated Caspase-3 and Bax/Bcl-2 compared with the OGD group, while BIRF siRNA transfection abolished this effect.

These results suggest that BIRF silencing impeded both the upregulation of GLT-1 and the induction of OGD tolerance by IPC.

### BIRF Knockdown Decreased the Expression of GLT-1 and Blocked the Induction of BIT In vivo

**Fig. 3:**
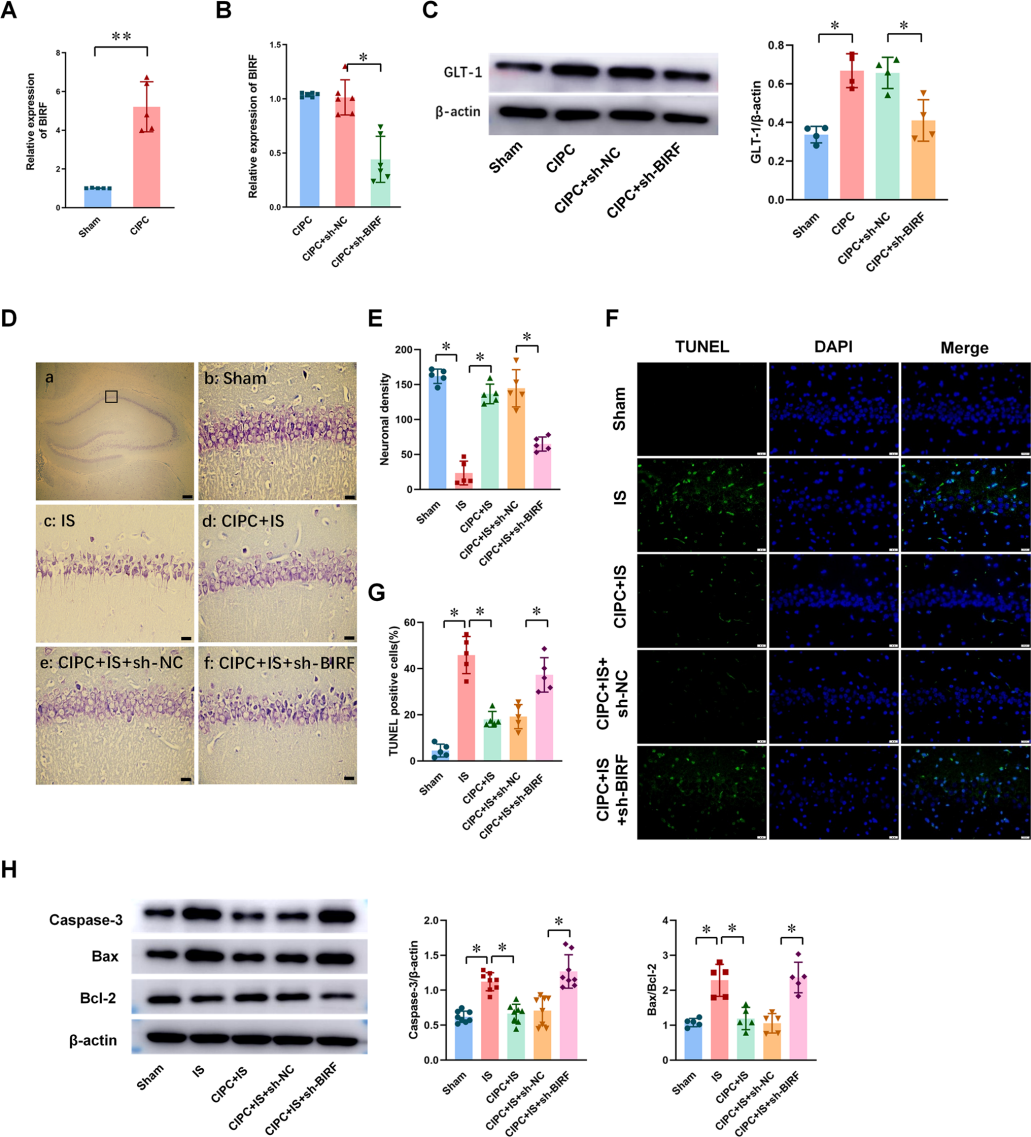
The effects of sh-BIRF on GLT-1 expression and BIT in vivo. **A** qRT-PCR analysis of the expression of BIRF after CIPC in hippocampal CA1 region in rats (*n* = 5). **B** qRT-PCR results showed that sh-BIRF preadministration decreased the expression of BIRF after CIPC (*n* = 6). **C** Western blot illustrates that sh-BIRF preadministration before CIPC decreased the expression of GLT-1 protein (*n* = 4). **D**,** E** Representative thionin staining photomicrographs show the effect of sh-BIRF on neuron survival (**D**) (scale bar is 200 μm in (a) and 20 μm in (b–f)) and neuron density (**E**) in CA1 hippocampus at 7 days after global brain ischemia in rats (*n* = 5). **F**,** G** TUNEL staining photomicrographs (**F**) (scale bar = 20 μm) and the percent of positive cells (**G**) show the effect of sh-BIRF on neuronal apoptosis in CA1 hippocampus at 7 days after global brain ischemia (*n* = 5). **H** The effect of sh-BIRF preadministration on caspase-3 (*n* = 8) and Bax/Bcl-2 (*n* = 5) in CA1 subfield in rats during the induction of BIT. Data are presented as the mean ± SD. *P < 0.05, **P < 0.01

We established a four-vessel occlusion model to simulate global brain ischemia in vivo. Compared with the sham group, BIRF expression in the hippocampal CA1 region increased in the CIPC group in rats (Fig. 3A).

Compared with the CIPC+sh-NC group (Fig. S3), markedly downregulated BIRF expression was observed in the CIPC+sh-BIRF group (Fig. 3B). Western blot analysis also showed that GLT-1 expression was increased after CIPC, while BIRF knockdown inhibited this effect (Fig. 3C). Thionin staining showed that neurons in the hippocampal CA1 region of the sham group were neatly arranged in 2–3 layers (Fig. 3D). Each cell had a complete cell contour, with round nuclei and clear nucleoli. A significant decrease in neuronal density occurred at 7 days after global brain ischemia for period of 8 min (IS group), manifested as neuronal destruction and loss, which was attenuated by CIPC treatment before IS (CIPC + IS group). However, in the CIPC+IS+sh-BIRF group, neuron injury was significantly aggravated, as shown by a disordered arrangement, nuclear pyknosis or cell disintegration, and decreased density of neurons (Fig. 3D, E). The results of TUNEL staining (Fig. 3F, G) and western blot (Fig. 3H) showed that severe ischemia could aggravate cell apoptosis, while CIPC could reduce neuronal apoptosis and induce BIT. However, pre-injection of sh-BIRF before ischemia interfered with CIPC-induced BIT, resulting in aggravate neuronal apoptosis. These findings were consistent with the results of thionin staining.

These results suggest that BIRF might regulate GLT-1 expression and participate in the induction of BIT during the process.

### The Interaction Between BIRF and miR-330-5p

**Fig. 4:**
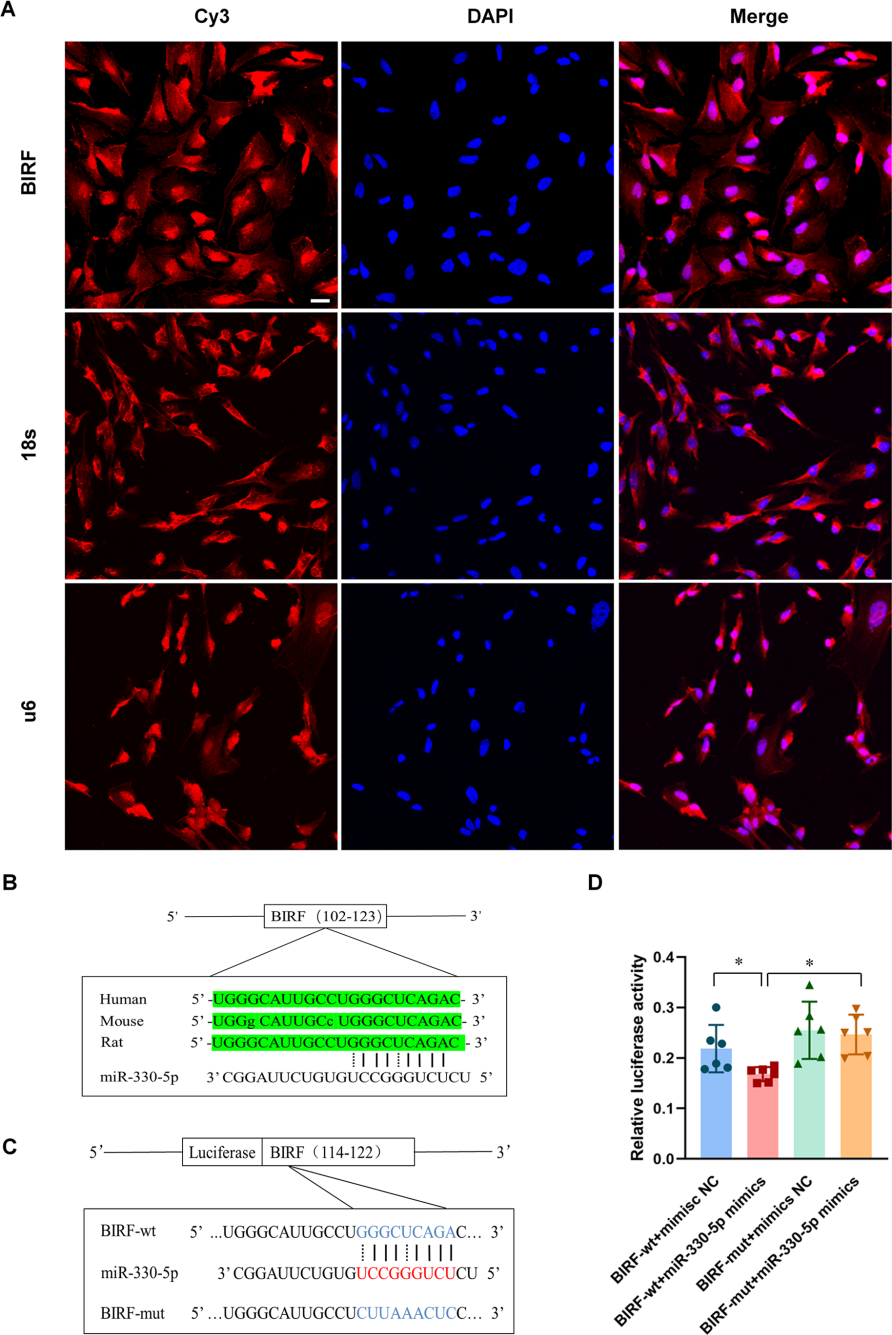
The interaction between BIRF and miR-330-5p.** A** FISH was conducted to explore the subcellular localization of BIRF in astrocytes (scale bar = 20 μm) (*n* = 3). **B** The binding site of BIRF and miR-330-5p is conserved between species. **C** Presentation of the putative binding site of miR-330-5p and BIRF (BIRF-wt), and the designed mutant sequence (BIRF-mut). **D** The relative luciferase activities of 293-T cells co-transfected BIRF-wt or BIRF-mut with miR-330-5p mimics or mimics negative control (*n* = 6). Data are presented as the mean ± SD. *P < 0.05

It is well known that the subcellular localization of lncRNAs impacts their function, and lncRNAs generally regulate the translation of target genes in the cytoplasm [32]. To explore whether BIRF acted as an endogenous sponge RNA to interact with miR-330-5p, we used RNA fluorescence in situ hybridization (FISH) to explore the subcellular localization of BIRF in astrocytes, and the result showed that BIRF was presented in both the cytoplasm and the nucleus (Fig. 4A). We found the sequence of BIRF on NONCODE (http://www.noncode.org/) and used sequence alignment on UCSC (http://genome.ucsc.edu/) to find that while full-length BIRF is poorly conserved across species, the binding site on miR-330-5p is conserved between species (Fig. 4B). To determine whether BIRF could interact with miR-330-5p, we compared the sequence of BIRF with that of miR-330-5p using the bioinformatics database RNAhybrid (https://bibiserv.cebitec.uni-bielefeld.de/rnahybrid/) and speculated on a putative binding site between BIRF and miR-330-5p (Fig. 4C). Furthermore, we performed dual-luciferase reporter gene analysis to confirm this binding site in 293-T cells (Fig. 4D). Luciferase assay revealed that miR-330-5p mimics could reduce the luciferase activity of BIRF-wt, but it had less effect on BIRF-mut. These results suggest that BIRF may directly bind to miR-330-5p.

### miR-330-5p Inhibited GLT-1 Expression and OGD Tolerance In vitro and In vivo

According to the bioinformatics prediction shown in Fig. 1, we found that miR-330-5p, BIRF, and GLT-1 constituted a ceRNA interaction net. We have explored the relationship between miR-330-5p and BIRF, and we will now proceed to investigate the effect of miR-330-5p on GLT-1 expression and OGD tolerance.

**Fig. 5:**
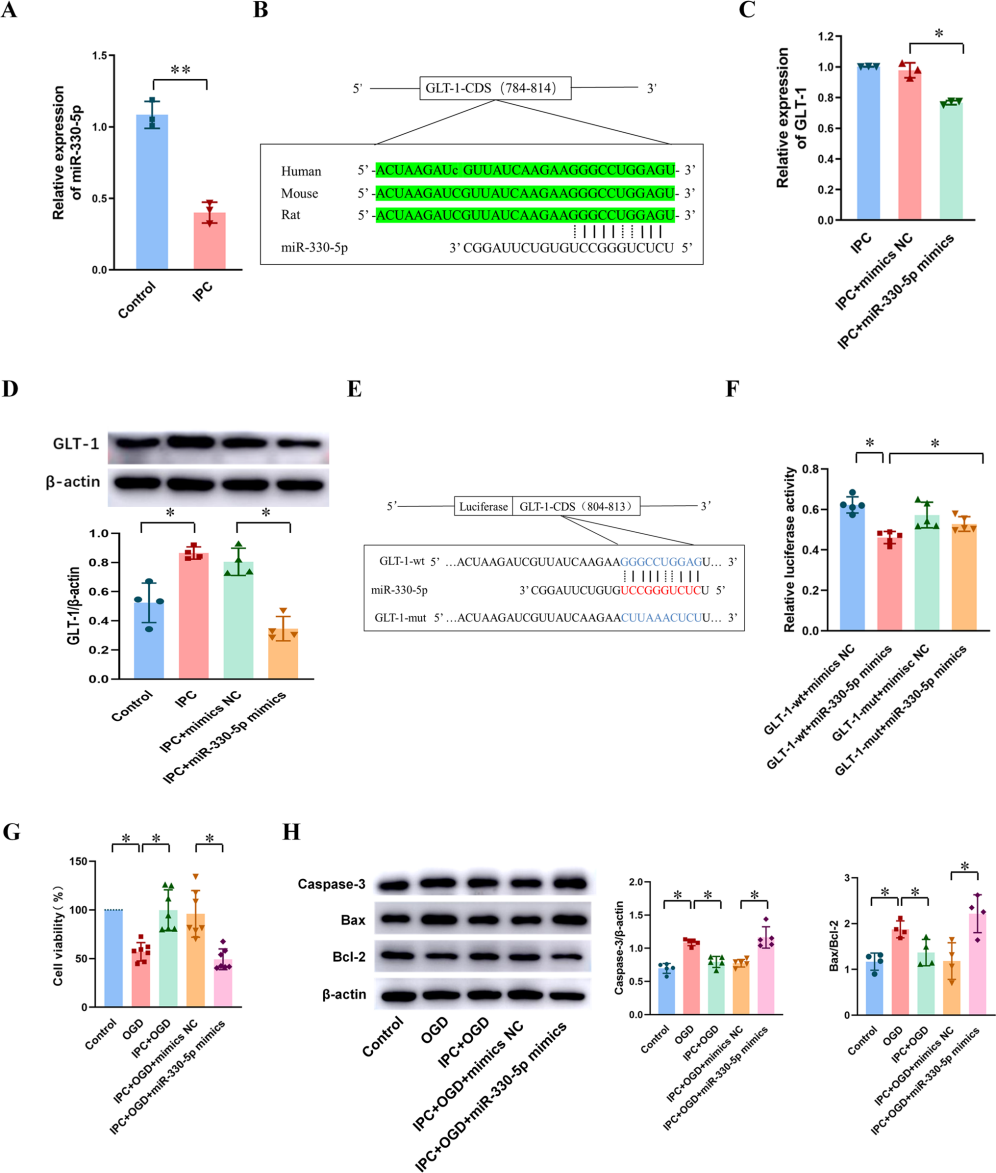
The effect of miR-330-5p on GLT-1 expression and OGD tolerance in vitro. **A** The expression of miR-330-5p in astrocytes after IPC (*n* = 3). **B** The binding site of GLT-1 and miR-330-5p is conserved between species. **C**, **D** The effect of miR-330-5p mimics on the expression of GLT-1 mRNA (**C**) (*n* = 3) and protein (**D**) (*n* = 4) in astrocytes. **E** Presentation of the putative binding site of miR-330-5p and GLT-1 (GLT-1-wt), and the designed mutant sequence (GLT-1-mut). **F** The relative luciferase activities of 293-T cells co-transfected GLT-1-wt or GLT-1-mut with miR-330-5p mimics or mimics NC (*n* = 5). **G** CCK-8 assay was used to investigate the cell viability of co-cultured neurons after astrocytes pretransfection with miR-330-5p mimics (*n* = 7). **H** The influence of miR-330-5p mimics on caspase-3 (*n* = 5), Bax/Bcl-2 (*n* = 4) expression in neurons. Data are presented as the mean ± SD. *P < 0.05, **P < 0.01

In this study, miR-330-5p was downregulated after IPC in co-cultured astrocytes (Fig. 5A). We found the sequence of GLT-1 on NCBI (https://www.ncbi.nlm.nih.gov/), and using sequence alignment on UCSC (http://genome.ucsc.edu/), we found that the binding site of GLT-1 and miR-330-5p is conserved between species (Fig. 5B). We next transfected astrocytes with miR-330-5p mimics to perform further investigation (Fig. S4), and the expressions both of GLT-1 mRNA and protein were inhibited after the transfection (Fig. 5C, D). Furthermore, RNAhybrid (https://bibiserv.cebitec.uni-bielefeld.de/rnahybrid/) provided a putative binding site between miR-330-5p and GLT-1-CDS (Fig. 5E), and dual-luciferase reporter gene analysis was performed to confirm GLT-1-CDS as a direct target of miR-330-5p in 293-T cells (Fig. 5F). Cells transfected with both GLT-1-wt and miR-330-5p mimics showed significantly reduced relative luciferase activity compared with other groups. Cell viability and apoptosis were detected by CCK 8 and western blot assays, respectively, and the results indicated that miR-330-5p mimics transfection resulted in decreased neuronal viability (Fig. 5G), increased the expressions of caspase-3 and Bax, and decreased the expression of Bcl-2 in neurons (Fig. 5H). Taken together, these results reveal that GLT-1 is a direct target of miR-330-5p and that miR-330-5p inhibits OGD tolerance in vitro.

**Fig. 6:**
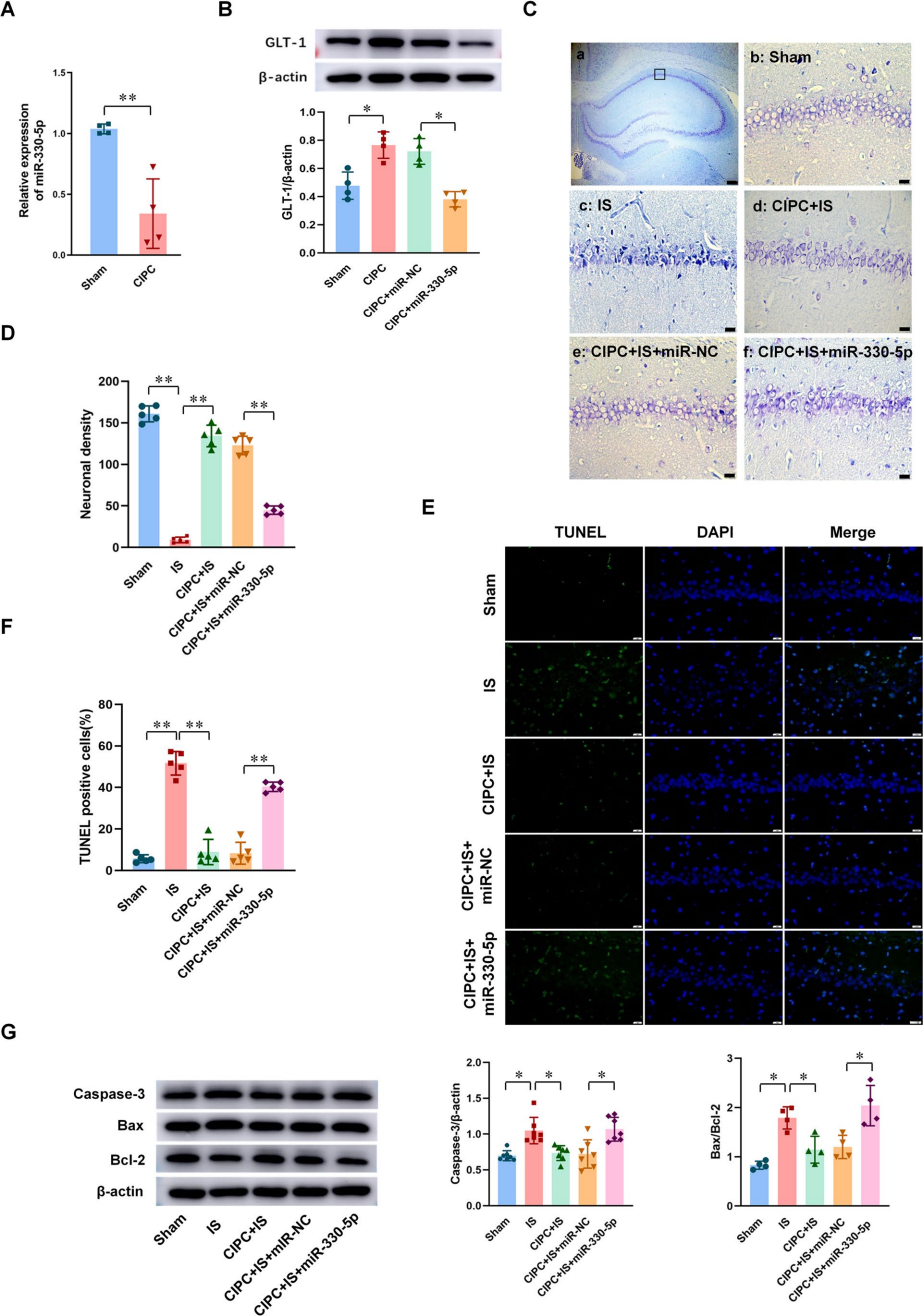
Overexpression of miR-330-5p inhibited GLT-1 expression and BIT in vivo. **A** The expression of miR-330-5p in hippocampal CA1 subfield in rats after CIPC (*n* = 4). **B** Western blot illustrates the effect of miR-330-5p on the expression of GLT-1 protein after CIPC (*n* = 4). **C**, **D** Representative thionin staining photomicrographs show the effect of miR-330-5p on neuronal survival (**C**) (scale bar is 200 μm in (a) and 20 μm in (b–f)) and neuron density (**D**) at 7 days in CA1 hippocampus after global brain ischemia in rats (*n* = 5). **E**, **F** TUNEL staining photomicrographs (**E**) (scale bar = 20 μm) and the percent of positive cells (**F**) show the effect of miR-330-5p on neuronal apoptosis at 7 days after global brain ischemia in hippocampal CA1 subfield (*n* = 5). **G** The effect of miR-330-5p on the expressions of caspase-3 (*n* = 7) and Bax/Bcl-2 (*n* = 4) in rats during the induction of BIT by western blot. Data are presented as the mean ± SD. *P < 0.05, **P < 0.01

We also attempted to explore the role of miR-330-5p in the pathogenesis of BIT in vivo. The expression of miR-330-5p in the hippocampal CA1 subfield was found to be downregulated in rats after CIPC (Fig. 6A). Overexpression of miR-330-5p (Fig. S5) resulted in decreased GLT-1 protein after CIPC (Fig. 6B), and the results of thionin staining (Fig. 6C, D) showed that BIT disappeared in rats preinjected with pAAV-miR-330-5p. The results of TUNEL staining (Fig. 6E, F) and the expression of apoptosis-related proteins (Fig. 6G) were consistent with the results of in vitro experiments. This indicates that miR-330-5p inhibited GLT-1 expression and BIT induced by CIPC in rats.

### BIRF Acts as a Molecular Sponge to Competitively Bind to miR-330-5p with GLT-1 mRNA

**Fig. 7:**
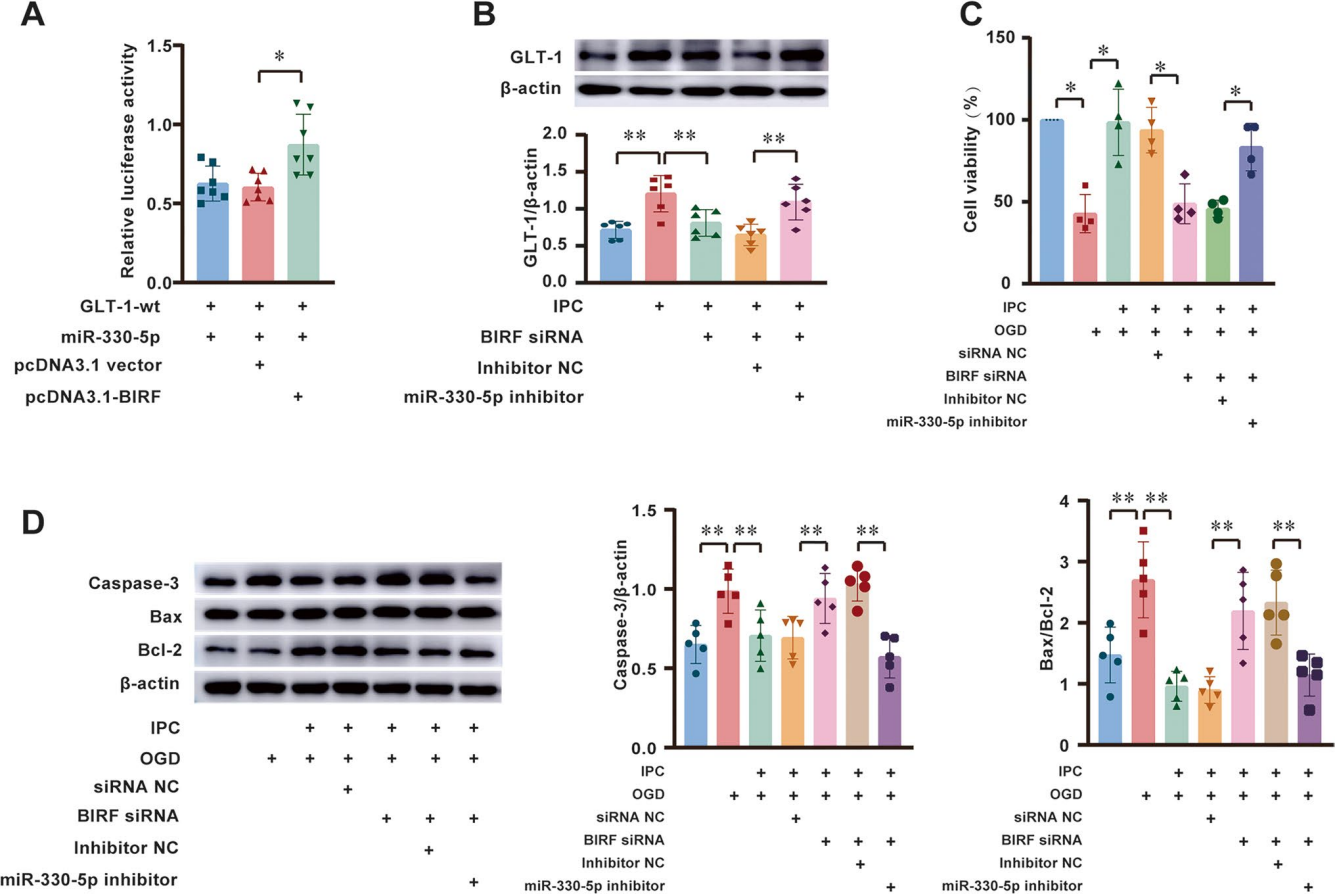
BIRF acts as a ceRNA in regulating GLT-1 expression by binding to miR-330-5p.** A** Dual luciferase results showed that pcDNA3.1-BIRF reversed the inhibitory effect of miR-330-5p mimics on GLT-1-wt in 293-T cell (*n* = 7). **B** The effect of miR-330-5p inhibitor on GLT-1 protein in astrocyte after transfection of BIRF siRNA (*n* = 6). **C**,** D** miR-330-5p inhibitor reversed the effect of BIRF siRNA on OGD tolerance (**C**) (*n* = 4) and the expressions of caspase-3 and Bax/Bcl-2 protein (**D**) (*n* = 5) in neurons. Data are presented as the mean ± SD. *P < 0.05, **P < 0.01

We performed a luciferase assay to investigate whether BIRF regulates GLT-1 by competitively binding to miR-330-5p in 293-T cells. We have demonstrated that miR-330-5p mimics could bind to GLT-1-wt and reduce its luciferase activity (Fig. 5F), and this effect was reversed after the BIRF overexpression by pcDNA3.1-BIRF. In other words, the fluorescence activity was restored by BIRF (Fig. 7A).

**Fig. 8:**
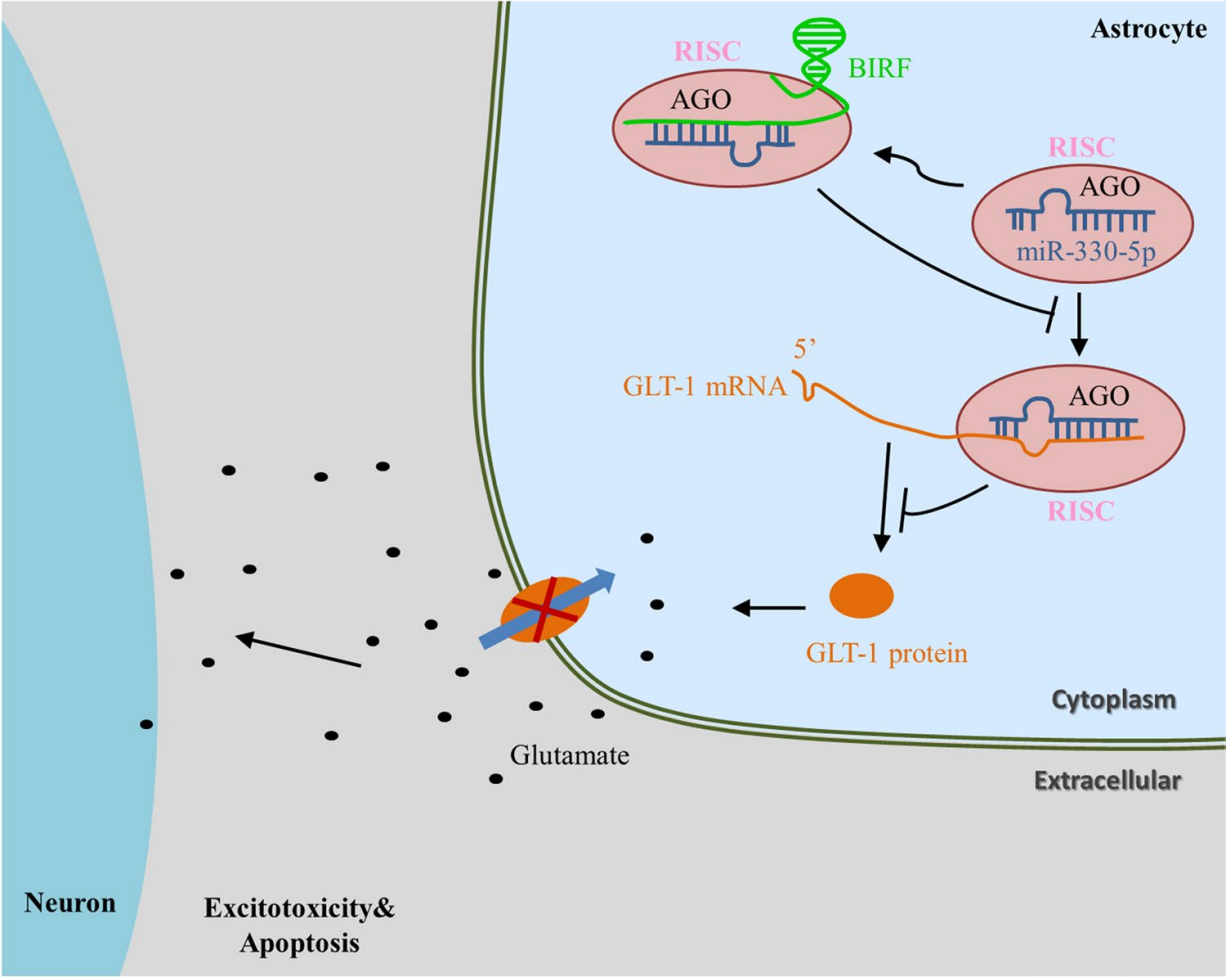
The schematic description of the mechanism of BIRF/miR-330-5p/GLT-1 axis in regulating BIT

In order to determine whether miR-330-5p inhibitor could reverse the regulatory effects of BIRF siRNA on GLT-1 expression and neuronal vitality, we transfected BIRF siRNA and the miR-330-5p inhibitor at the same time. As shown in Fig. 7B, the miR-330-5p inhibitor could reverse the effect of BIRF siRNA on GLT-1 expression in astrocytes. The results of the CCK8 assay showed that BIRF siRNA could inhibit IPC-induced OGD tolerance of neurons. However, co-transfection with miR-330-5p inhibitor could reverse this effect (Fig. 7C). Moreover, the results of western blot showed that increased expression of apoptotic-related proteins induced by BIRF siRNA in neurons was reversed following co-transfection with miR-330-5p inhibitor in astrocytes (Fig. 7D). These results imply that BIRF regulates GLT-1 expression and OGD tolerance through miR-330-5p (Fig. 8).

## Discussion

Our previous studies have proved that CIPC can induce BIT by upregulating GLT-1 expression [20–23]. Nevertheless, the associated underlying mechanisms in CIPC have not previously been comprehensively explored. The present study seeks to explore the ceRNA regulatory network between BIRF, miR-330-5p, and GLT-1 in the induction of BIT by CIPC.

In recent years, the interest in lncRNAs is on the rise in the epigenetic view of physiological and pathological processes. LncRNAs are abundant in the mammalian brain and play important roles in many neurodegenerative diseases, such as Alzheimer’s disease [33], Parkinson’s disease [34], and ischemic stroke [35]. Many aberrantly expressed lncRNAs were identified both in animal brain and in patients’ blood after stroke [36, 37]. lncRNA C2dat1 regulated CaMKIIδ expression, which promoted neuronal survival from cerebral ischemia through the NF-κB signaling pathway [38]. Silencing of lncRNA GAS5 suppressed neuron apoptosis in ischemic stroke through inhibiting DNMT3B-dependent methylation of MAP4K4 [39]. In this study, we identified 323 downregulated lncRNAs and 329 upregulated lncRNAs through RNA-seq in the IPC group compared with the control group. Among the identified lncRNAs, we confirmed that BIRF was significantly upregulated, and the knockdown of BIRF could significantly inhibit OGD tolerance. As expected, both in vitro and in vivo experiments revealed that BIRF knockdown decreased GLT-1 expression and impeded the induction of BIT by CIPC. To the best of our knowledge, we are the first to identify BIRF as a novel lncRNA involved in the induction of BIT and as a participant in the process through regulating GLT-1 expression.

A great number of studies have shown that lncRNA plays an important role in cerebral ischemic stroke through the ceRNA mechanism. LncRNAs are abundant in miRNA-binding sites, which can competitively adsorb miRNA and block or reduce the inhibition effect of miRNA on its target genes, thereby promoting the expression of the genes [12]. In cerebral ischemic stroke, lncRNA KCNQ1OT1 promotes I/R-induced autophagy and decreases cell viability via the miR-200a/FOXO3/ATG7 pathway [40]. LncRNA MALAT1 silencing protects MA-C cell survival from cerebral ischemia-reperfusion injury through the miR-145/AQP4 pathway [41]. Using the sequencing results library construction and sequence alignment produced by Sinotech Genomics company, we predicted that miR-330-5p might bind to BIRF, and the direct binding site between BIRF and miR-330-5p was confirmed by dual luciferase assay. The role of miR-330-5p in cerebral ischemic stroke has not been reported yet, but researchers have found that miR-330-5p participates in myocardial I/R injury [29]. MiR-330-5p inhibits NLRP3 inflammasome-mediated myocardial I/R injury by targeting TIM3, and inhibition of miR-330-5p significantly aggravated myocardial I/R injury, resulting in increased infarct volume and more severe cardiac dysfunction [29]. miR-330-5p is also reported to modulate inflammation and cell apoptosis. In atherosclerosis, circ_0065149 alleviates oxidized low-density lipoprotein-induced apoptosis and inflammation by targeting miR-330-5p [42]. In addition, most existing studies have shown that miR-330-5p plays an important role in the regulation of metastatic invasion and epithelial-mesenchymal transformation in tumors [43]. In the present study, we investigated the role of miR-330-5p in BIT. We observed that the miR-330-5p expression was reduced during the induction of OGD tolerance, and overexpression of miR-330-5p decreased GLT-1 expression and inhibited the induction of OGD tolerance, resulting in the decline of neuronal viability and aggravation of neuron apoptosis. The RNAhybrid database predicted that miR-330-5p might target the GLT-1 CDS region, and dual-luciferase reporter gene analysis demonstrated that miR-330-5p can directly target GLT-1 to regulate its expression. The results in vivo were consistent with those in vitro. These results implied that miR-330-5p might regulate both OGD tolerance and BIT via the GLT-1 pathway. To the best of our knowledge, our study is the first to demonstrate that GLT-1 is a target of miR-330-5p during the induction of BIT. We also established that overexpression of BIRF could bind to miRNAs’ target sites and prevented miR-330-5p from directly binding to GLT-1 mRNA. In addition, the miR-330-5p inhibitor could reverse the effects of BIRF siRNA on GLT-1 expression and OGD tolerance. These results provided sufficient evidence for BIRF as a sponge for miR-330-5p, thereby regulating the expression of GLT-1. In a word, the BIRF/miR-330-5p/GLT-1 axis participated in OGD tolerance/BIT induction.

Some researchers have found that GLT-1 can be regulated by miRNAs to participate in cerebral ischemic stroke. miR-124 upregulates glutamate transporter-1 in astrocytes via the Akt and mTOR signaling pathway after cerebral ischemic stroke [44]. Magnesium lithospermate B is able to protect the rat brain from excitatory neurotoxicity during I/R through the regulation of miR-107/GLT-1 pathway [45]. It has been shown that GLT-1 upregulation can increase glutamate uptake, thereby protecting astrocytes and neurons from hypoxia-induced apoptosis. For example, Emodin promoted Bcl-2 and GLT-1 expression to inhibit neuronal apoptosis and ROS generation via ERK-1/2 signaling pathway [46]. Orexin-A promotes glutamate uptake via the OX1R/PKCα/ERK1/2/GLT-1 pathway in astrocytes and protects co-cultured neurons and astrocytes against apoptosis in anoxia/hypoglycemic injury in vitro [47]. In the present study, BIRF or miR-330-5p regulates GLT-1 expression and neuron apoptosis. In conclusion, our findings demonstrated for the first time that BIRF acts as an endogenous miR-330-5p sponge to inhibit its activity, resulting in the increased expression of GLT-1. Importantly, the binding sites between BIRF and miR-330-5p, miR-330-5p, and GLT-1 are conserved among species, which might be a new therapeutic approach to treating cerebral ischemic stroke.

## Supplementary Information

Below is the link to the electronic supplementary material.

Supplementary file1 (PDF 439 KB)

Table S7 GO enrichment and Pathway analysis of mRNAs from the ceRNA network. (XLSX 113 KB)

Supplementary file3 (PDF 584 KB)

## Data Availability

The result of lncRNA high-throughput sequencing (RNA-seq) have been uploaded to GEO database (GSE185931, https://www.ncbi.nlm.nih.gov/geo/query/acc.cgi?acc=GSE185931), and released on November 1, 2021. The other data generated or analyzed during this study are included in this published article and its supplementary information files.
