# Supplementary material for: LncRNA BIRF Promotes Brain Ischemic Tolerance Induced By Cerebral Ischemic Preconditioning Through Upregulating GLT-1 via Sponging miR-330-5p
Source: Mol Neurobiol. 2022 Apr 22;59(7):3996–4014. doi: 10.1007/s12035-022-02841-3 (PMC9167204; doi:10.1007/s12035-022-02841-3)
Supplement: Supplementary file 1 — Supplementary file1 (PDF 439 KB) [file 12035_2022_2841_MOESM1_ESM.pdf]

LncRNA BIRF Promotes Brain Ischemic Tolerance Induced By Cerebral Ischemic Preconditioning Through Upregulating GLT-1 Via Sponging miR-330-5p

Shichao Li[1] · Lingyan Zhang[1] · Jiajie Lin[2] · Achou Su[1] · Xiyun Liu[1] · Jingge Zhang[1] · Xiaohui Xian[1] · Yuyan Hu[1] · Wenbin Li[1] ·Shaoguang Sun[2] · Min Zhang[1]

[1] Key Laboratory of Critical Disease Mechanism and intervention of Hebei Province, Hebei Medical University, Department of Pathophysiology, Shijiazhuang, China

[2] Key Laboratory of Medical Biotechnology of Hebei Province, Cardiovascular Medical Science Center, Hebei Medical University, Department of Biochemistry and Molecular Biology, Shijiazhuang, China

✉ Shaoguang Sun (co-corresponding author)

sunshaoguang00@163.com

✉ Min Zhang (corresponding author)

hebmuzhangmin@163.com

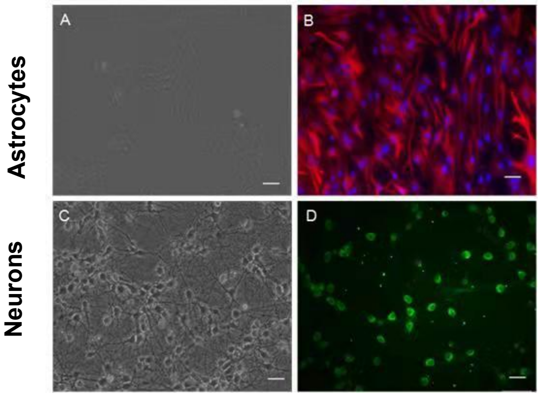

Fig S1. Representative photomicrographs of astrocyte marker GFAP and neuron marker NSE. **(A)** and **(B)** Astrocytes and astrocyte marker GFAP. **(C)** and **(D)** Neurons and neuron marker NSE (scale bar = 20 μm).

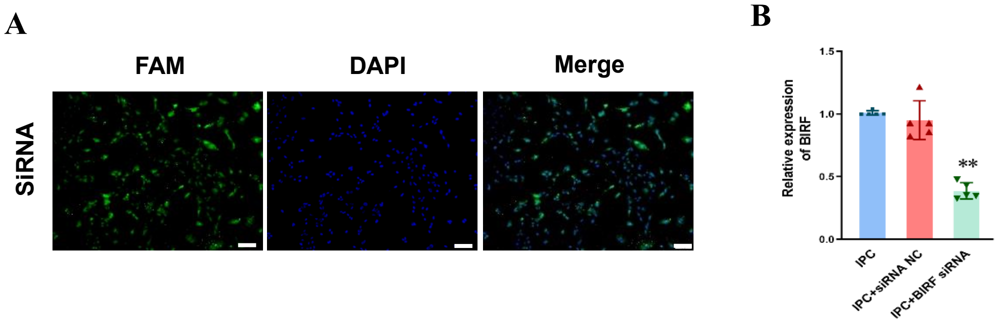

Fig S2. Validation of BIRF siRNA transfection efficiency. **(A)** Representative photomicrographs of siRNA NC-FAM transfection in astrocytes (scale bar = 100μm). **(B)** qRT-PCR result shows that BIRF siRNA pre-transfection in astrocytes decreased the expression of BIRF after IPC (n=5). $**P < 0.01$ vs. IPC+siRNA NC group.

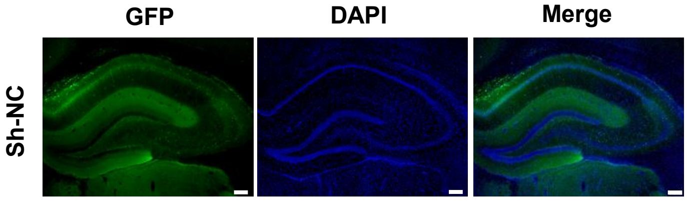

Fig S3. Representative photomicrographs of pAAV-sh-NC-GFP infection of the lateral ventricle (scale bar = 200 μm).

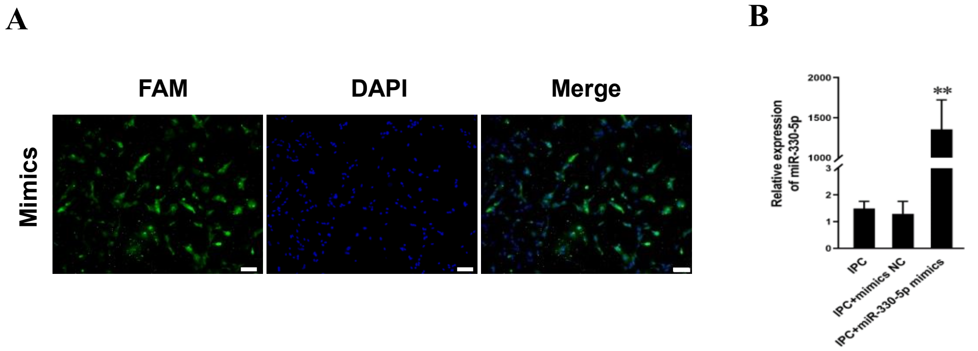

Fig S4. Validation of miR-330-5p mimics transfection efficiency. **(A)** Representative photomicrographs of mimics NC-FAM transfection in astrocytes (scale bar = 100μm). **(B)** qRT-PCR result shows that miR-330-5p mimics pre-transfection in astrocytes increased the expression of miR-330-5p after IPC (n=3). ***P* < 0.01 vs. IPC+mimics NC group.

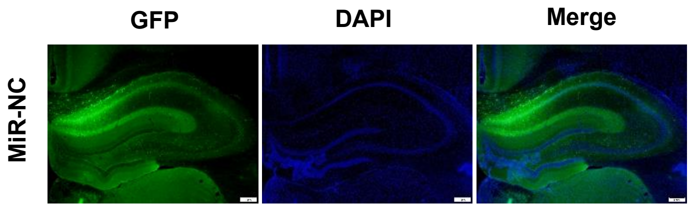

Fig S5. Representative photomicrographs of pAAV-miR-NC-GFP infection of the lateral ventricle (scale bar = 200 μm).

Table S1 The sequences of pAAV-sh-RNA and pAAV-miR

ID	pAAV-sh-RNA or pAAV-miR sequences (5'~3')
AAV-NC	TTCTCCGAACGTGTCACGT
NONRATT009133.2	GGCTCAGACATACACAGTT
rno-miR-330-5p	TCTCTGGGCCTGTGTCTTAGGC

Table S2 The siRNA sequences of lncRNAs

ID	siRNA sequences (5'~3')
Negative control	Sense: UUCUCCGAACGUGUCACGUTT<br>Antisense: ACGUGACACGUUCGGAGAATT
NONRATT010451.2	Sense: GGAAGGGAGGCAGCCCUAATT<br>Antisense: UUAGGGCUGCCUCCCUUCCTT
NONRATT009133.2	Sense: GGCUCAGACAUACACAGUUTT<br>Antisense: AACUGUGUAUGUCUGAGCCTT
NONRATT010720.2	Sense: GGUGUUGGCUUGCUGGUAATT<br>Antisense: UUACCAGCAAGCCAACACCTT
NONRATT029757.2	Sense: GCAGCAGGACUAACUGCUUTT<br>Antisense: AAGCAGUUAGUCCUGCUGCTT

Table S3 The mimics sequences of miRNAs

ID	miRNAs mimics sequences (5'~3')
Negative control	Sense: UUCUCCGAACGUGUCACGUTT<br>Antisense: ACGUGACACGUUCGGAGAATT
rno-miR-22-3p	Sense: AAGCUGCCAGUUGAAGAACUGU<br>Antisense: AGUUCUUCAACUGGCAGCUUUU
rno-miR-330-5p	Sense: UCUCUGGGCCUGUGUCUUAGGC<br>Antisense: CUAAGACACAGGCCCAGAGAUU
rno-miR-347	Sense: UGUCCCUCUGGGUCGCCCA<br>Antisense: GGCGACCCAGAGGGACAUU

Table S4 The inhibitor sequences of miRNAs

ID	miRNAs inhibitor sequences (5'~3')
Inhibitor N.C.	CAGUACUUUUGUGUAGUACAA
rno-miR-330-5p	GCCUAAGACACAGGCCCAGAGA

Table S5 The primer sequences of lncRNAs and Slc1a2

ID	Primer sequences (5'~3')
NONRATT001933.2	F: AATCGGCATAGCGTGTTTTC<br>R: CACTCAGTCCTCACACAGCA
NONRATT021426.2	F: GAGGCAAGCCAAGTGATCATTAA<br>R: GCAGCAAGACACCTTCCATTTT
NONRATT010720.2	F: AACCAGGCATGTTCAGTTTTG<br>R: GGCTACCACAGGGACTTTCATA
NONRATT009133.2	F: AAGGAACCACCCTACACTGAGA<br>R: GGGATTGCCTTGGAGTATAAGA
NONRATT010451.2	F: TGTGGCATCAAAGAGAAGGTTA<br>R: GGGGTATAGATGTGGAAGATGG
NONRATT026094.2	F: TGTCCCTCCCCCTCTTATTT<br>R: AAGTCCCTGGAGGTGATCTG
NONRATT029757.2	F: GTCATGCAAACTGGCAAAAGT<br>R: GACCCAGTTTTTCCAGGAGACC
Slc1a2 (GLT-1)	F: GTGGACTGGCTGCTGGATA<br>R: GTGGTTCTTCGTGTCGTCATAA
GAPDH	F: GCCAAAAGGGTCATCATCTCTG<br>R: CATGCCAGTGAGCTTCCCGT

Table S6 The primer sequences of miRNA

ID	Lot. NO.
rno-miR-330-5p	Cat# RmiRQP0418
U6	Cat# RmiRQP9003

Table S7 GO enrichment and Pathway analysis of mRNAs from the ceRNA network. (Excel).
