# Supplementary material for: LncRNA BIRF Promotes Brain Ischemic Tolerance Induced By Cerebral Ischemic Preconditioning Through Upregulating GLT-1 via Sponging miR-330-5p
Source: Mol Neurobiol. 2022 Apr 22;59(7):3996–4014. doi: 10.1007/s12035-022-02841-3 (PMC9167204; doi:10.1007/s12035-022-02841-3)
Supplement: Supplementary file 3 — Supplementary file3 (PDF 584 KB) [file 12035_2022_2841_MOESM3_ESM.pdf]

Figure 2

GLT-1

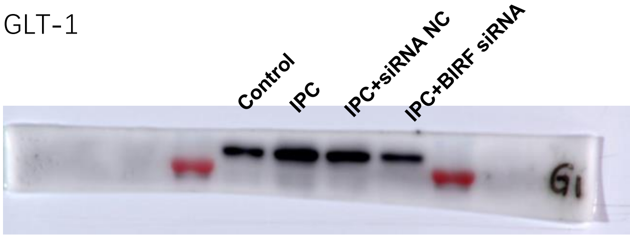

β-actin

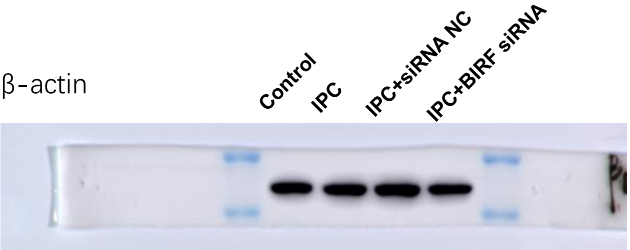

Caspase-3

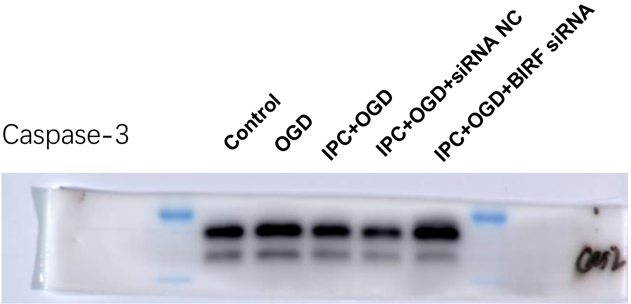

Bax

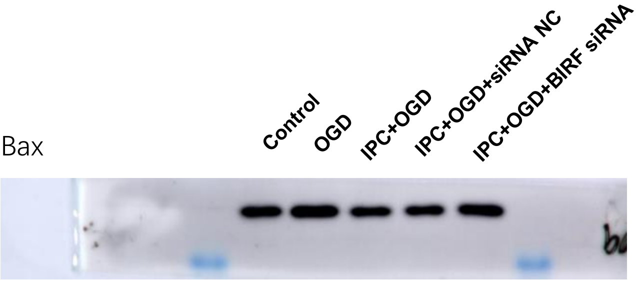

Bcl-2

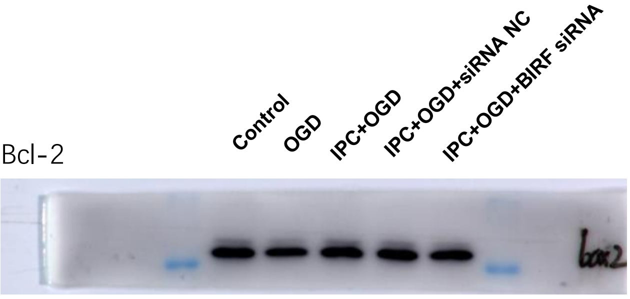

β-actin

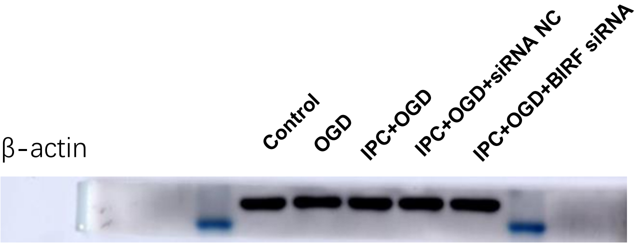

Figure 3

GLT-1

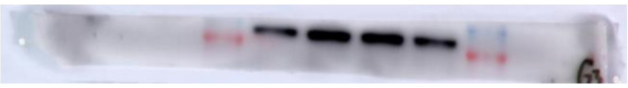

β-actin

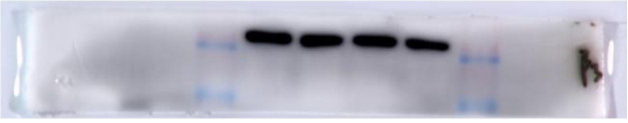

Caspase-3

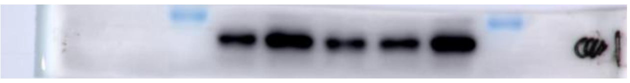

Bax

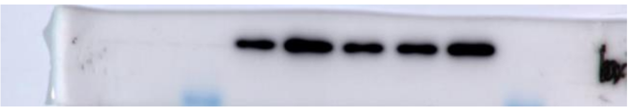

Bcl-2

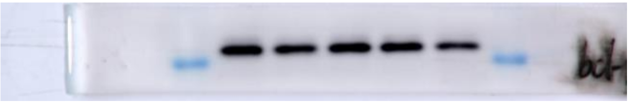

β-actin

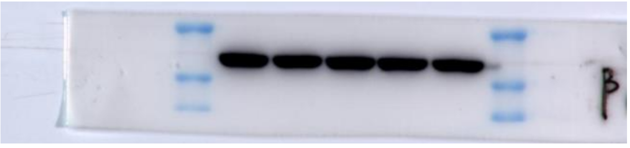

Figure 5

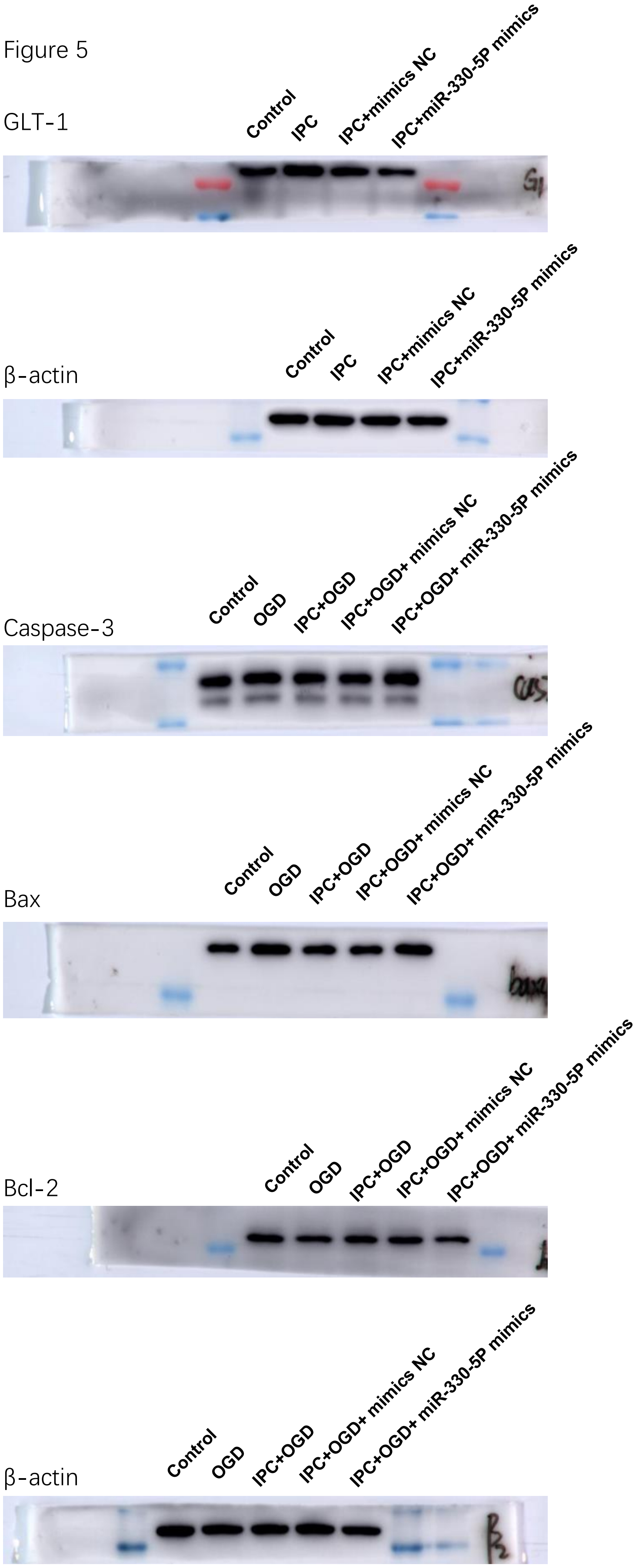
GLT-1
Control
IPC
IPC+mimics NC
IPC+miR-330-5P mimics
β-actin
Control
IPC
IPC+mimics NC
IPC+miR-330-5P mimics
Caspase-3
Control
OGD
IPC+OGD
IPC+OGD+ mimics NC
IPC+OGD+ miR-330-5P mimics
Bax
Control
OGD
IPC+OGD
IPC+OGD+ mimics NC
IPC+OGD+ miR-330-5P mimics
Bcl-2
Control
OGD
IPC+OGD
IPC+OGD+ mimics NC
IPC+OGD+ miR-330-5P mimics
β-actin
Control
OGD
IPC+OGD
IPC+OGD+ mimics NC
IPC+OGD+ miR-330-5P mimics

Figure 6

GLT-1

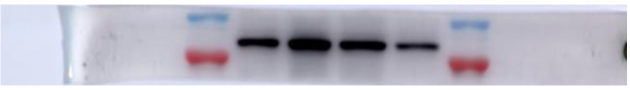

β-actin

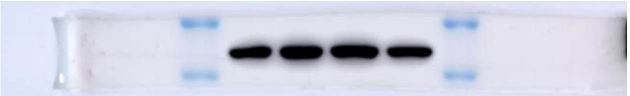

Caspase-3

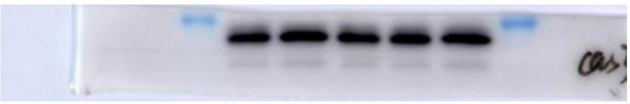

Bax

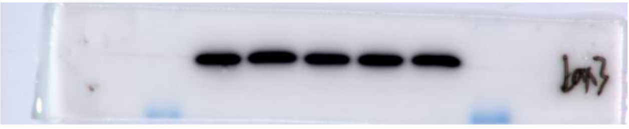

Bcl-2

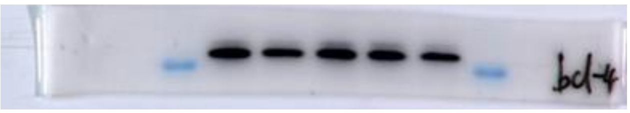

β-actin

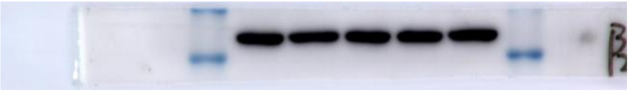

Figure 7

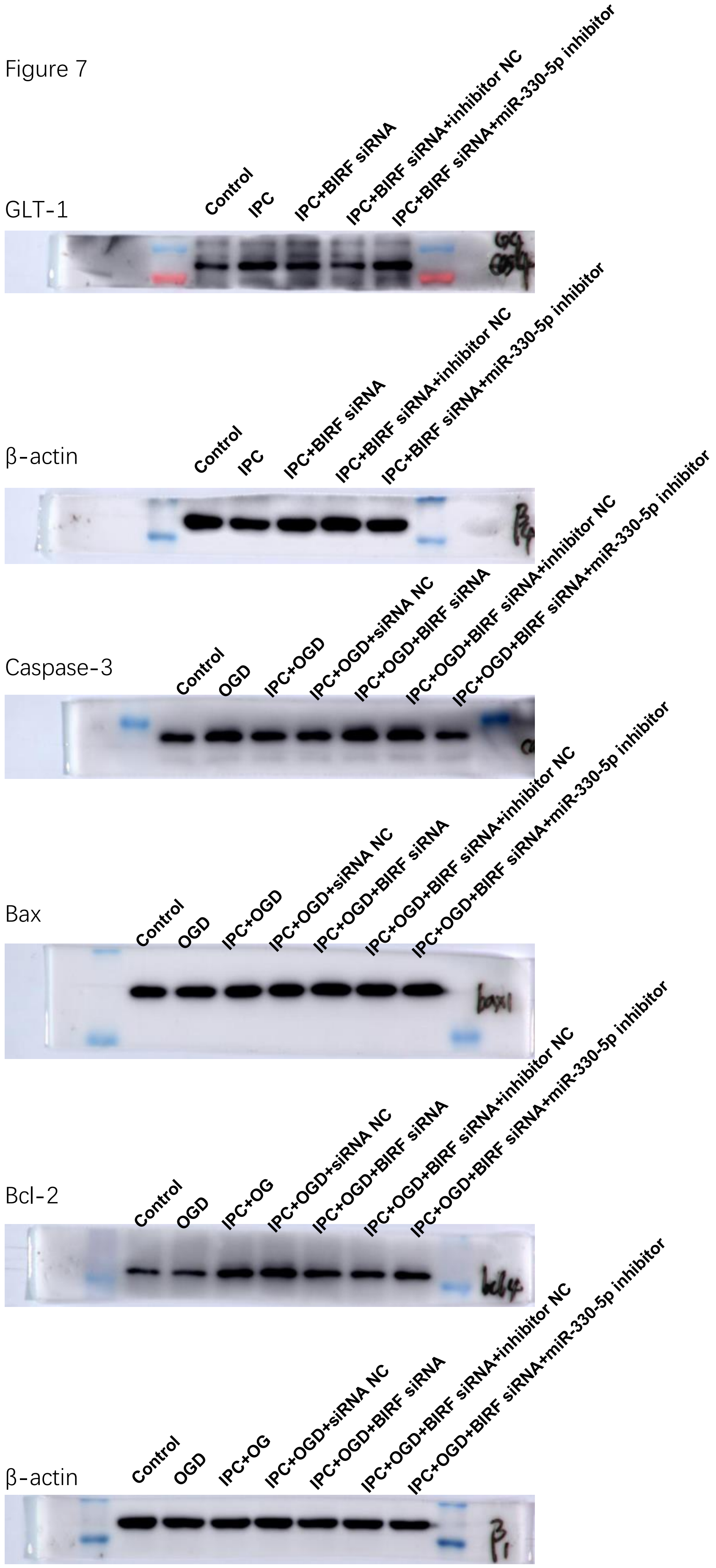
GLT-1
Control
IPC
IPC+BIRF siRNA
IPC+BIRF siRNA+inhibitor NC
IPC+BIRF siRNA+miR-330-5p inhibitor
β-actin
Control
IPC
IPC+BIRF siRNA
IPC+BIRF siRNA+inhibitor NC
IPC+BIRF siRNA+miR-330-5p inhibitor
Caspase-3
Control
OGD
IPC+OGD
IPC+OGD+siRNA NC
IPC+OGD+BIRF siRNA
IPC+OGD+BIRF siRNA+inhibitor NC
IPC+OGD+BIRF siRNA+miR-330-5p inhibitor
Bax
Control
OGD
IPC+OGD
IPC+OGD+siRNA NC
IPC+OGD+BIRF siRNA
IPC+OGD+BIRF siRNA+inhibitor NC
IPC+OGD+BIRF siRNA+miR-330-5p inhibitor
Bcl-2
Control
OGD
IPC+OG
IPC+OGD+siRNA NC
IPC+OGD+BIRF siRNA
IPC+OGD+BIRF siRNA+inhibitor NC
IPC+OGD+BIRF siRNA+miR-330-5p inhibitor
β-actin
Control
OGD
IPC+OG
IPC+OGD+siRNA NC
IPC+OGD+BIRF siRNA
IPC+OGD+BIRF siRNA+inhibitor NC
IPC+OGD+BIRF siRNA+miR-330-5p inhibitor
